# Natural Antisense Transcripts in Plants: A Review and Identification in Soybean Infected with *Phakopsora pachyrhizi* SuperSAGE Library

**DOI:** 10.1155/2013/219798

**Published:** 2013-06-26

**Authors:** Suzana de Aragão Britto-Kido, José Ribamar Costa Ferreira Neto, Valesca Pandolfi, Francismar Corrêa Marcelino-Guimarães, Alexandre Lima Nepomuceno, Ricardo Vilela Abdelnoor, Ana Maria Benko-Iseppon, Ederson Akio Kido

**Affiliations:** ^1^Federal University of Pernambuco (UFPE), Department of Genetics, Recife, PE, Brazil; ^2^Embrapa Soybean, Rod. Carlos João Strass, Distrito de Warta, Caixa Postal 231, 86.001-970 Londrina, PR, Brazil

## Abstract

Natural antisense ranscripts (NAT) are RNA molecules complementary to other endogenous RNAs. They are capable of regulating the expression of target genes at different levels (transcription, mRNA stability, translation, etc.). Such a property makes them ideal for interventions in organisms' metabolism. The present study reviewed plant NAT aspects, including features, availability and genesis, conservation and distribution, coding capacity, NAT pair expression, and functions. Besides, an *in silico* identification of NATs pairs was presented, using deepSuperSAGE libraries of soybean infected or not with *Phakopsora pachyrhizi*. Results showed that around 1/3 of the 77,903 predicted *trans*-NATs (by PlantsNATsDB database) detected had unitags mapped in both sequences of each pair. The same 1/3 of the 436 foreseen *cis*-NATs showed unitags anchored in both sequences of the related pairs. For those unitags mapped in NAT pairs, a modulation expression was assigned as upregulated, downregulated, or constitutive, based on the statistical analysis (*P* < 0.05). As a result, the infected treatment promoted the expression of 2,313 *trans*-NATs pairs comprising unitags exclusively from that library (1,326 pairs had unitags only found in the mock library). To understand the regulation of these NAT pairs could be a key aspect in the ASR plant response.

## 1. Introduction

Transcriptomics, bioinformatics, and high-throughput DNA sequencing have increased our understanding of global plant systems in responses and adaptations to many conditions, including a number of stresses [1]. Those analyses and the high amount of available information from several tissues and organisms allow a deep insight regarding the expression patterns of different genomes. A large proportion of prokaryotic and eukaryotic genomes is transcribed from both positive and negative strands of DNA and thus may generate overlapping sense and antisense transcripts, qualified as natural antisense transcripts (NAT). The first report showing a transcript with complementary base pairing in relation to another transcript was observed with virus studies. Bøvre and Szybalski [2] found that the central b2 region in the coliphage *λ* genome could produce two opposite oriented mRNAs, one originating on the plus strand and the other on the minus strand, that partially overlap with each other. Thereafter similar transcriptional events were identified in prokaryotes [3] and eukaryotes [4]. In plants, the first report covered *cis-*NATs coding SRO5 and P5CDH, involved in the regulation of salt tolerance through RNAi pathway in *Arabidopsis thaliana* [5]. NATs have been shown to regulate the expression of their target genes in different levels, including transcription, messenger RNA processing, splicing, cellular transport, and translation [6], adding a new complexity to the transcriptome analysis. Such a regulatory potential represents a fresh target for interferences at the cellular level, and this potential can be reached especially when the high-throughput sequencing technologies [7] are associated in transcriptomics with optimized bioinformatic analysis. In spite of the increasing importance of this transcript class, plant information availability is still low when compared to mammals and unicellular eukaryotes. Despite this, Chen et al. [8] developed a genome-scale computational pipeline to identify NATs in plant species; the database displays *in silico* predicted *cis-* and *trans-*NATs for 69 plant species, including soybean [*Glycine max* (L.) Merr.], an important legume, widely cultivated and consumed around the world. This paper presents the state of the art on the NAT (*cis* and *trans*) plant expression and activity, together with a NAT-related transcripts' expression analysis, based on SuperSAGE unitags (26 bp) from soybean leaves inoculated with *Phakopsora pachyrhizi*, the etiological agent of the Asian soybean rust (ASR), as compared with the negative control (noninfected). SuperSAGE is a well-established technique capable of generating a comprehensive transcriptional profile, especially when in association with high-throughput DNA sequencing [9–11]. This effort represents a useful combination for the gene regulation study by helping to identify and select important molecular targets in plant response to the applied stress.

In regard to the distribution of NATs, there are divergent opinions. Some authors report a random pattern of NATs loci across different genomes, in both mammals [12] and plants [13, 14], while others indicate the opposite [15]. According to Jouannet and Crespi [16], both ends of protein-coding genes have susceptibility for NAT occurrence, but they are not uniformly distributed. Sun et al. [17] reported the existence of hotspots in the 1.5 kb downstream positions of sense genes, while, for Seila and Sharp [18], an antisense transcription is enriched 250 nucleotides upstream from the transcription start site.

## 2. NAT Features, Availability in Plants, and Genesis

NATs are RNA molecules that are complementary to other transcripts. They are transcribed from DNA strands that are considered to be antisense. There are two main NAT categories: *cis-*NATs and *trans-*NATs. *cis-*NATs are antisense RNA transcribed from a single locus, due to the existence of a physical overlap of two genes in different strands, usually having specific targets (one-to-one style) [13]. On the other hand, *trans-*NATs are RNAs transcribed from different loci, displaying imperfect complementarities; therefore, they are able to aim at many sense targets forming complex regulation networks [19].

For *cis-*NATs, the transcription of the convergent gene occurs due to the presence of two close and antiparallel promoters, located in the same DNA molecule. This configuration has been reported by several groups [14, 20, 21]. Studies estimate that approximately 15% of the gene loci in rat have genes overlapping in opposite directions [22]. In humans, this conjecture reaches about 20% of the total gene loci [23]. The number changes depending on the methodology applied (predefined parameters and software used). In plants, the estimates are around 7% in rice [*Oryza sativa*, [24]] and 9% in Arabidopsis [13], representing a reduced number of sense-antisense transcript pairs, when compared with mammals and unicellular eukaryotes. Nevertheless, in almost all (99%) NAT pairs in Arabidopsis genome, the overlapping region includes exon sequences, except for a few of them in which one strand is entirely transcribed from the intronic sequences of the other gene [13]. Still regarding Arabidopsis, the majority of the overlapping gene pairs (956 pairs among the 1,083 identified) are organized with their overlaid regions comprising between 1 and 2,820 bp (mean length of 431 bp) [14]. Furthermore, in genomes anchoring *cis-*NAT pairs (sense and antisense transcripts), five different configurations were observed. They were characterized according to their relative orientation and degree of overlap. Thus, “tail-to-tail or convergent” are overlapping genes connected via their 3′ UTRs, “head-to-head or divergent” constitute overlapping genes connected via their respective 5′ UTRs, “fully overlapping” consisted of two genes in opposite strands, “nearby head-to-head” when the 5′ UTR of one gene is close to the 5′ UTR of the other gene, and finally “nearby tail-to-tail” with the 3′ UTR from one gene being near to the 3′ UTR of the other [25]. Among these, the most frequently found from plants [13, 14] to humans [26, 27] is the tail-to-tail type. 

Almost all studies addressing NATs focuses mainly on *cis-*NATs since they are easier to identify. However, there are reports concerning *trans-*NATs as well. Røsok and Sioud [28] revealed that about 50% of the cloned dsRNAs from human normal mammary epithelial and breast cancer cells are *trans-*NATs. With the use of ESTs, which significantly increased analysis coverage, long *trans-*NATs were also identified in animals, such as *Mus musculus* (mouse), *Rattus norvegicus* (rat), *Bos taurus* (cattle), *Canis lupus familiaris* (dog), *Gallus gallus* (chicken), *Danio rerio* (zebrafish), *Drosophila melanogaster* (fly), *Caenorhabditis elegans* (worm), and *Ciona intestinalis* (sea squirt) [29]. The highest percentage of transcriptional units (TUs) involved in *trans-*NATs, among all TUs, was 4.13% (sea squirt). In plants, Wang et al. [30], analyzing the Arabidopsis genome, identified 1,320 putative *trans-*NAT pairs. 

More robust data covering *cis-* and *trans-*NATs, in plants, were disclosed only after 2012. Chen et al. [8] developed a plant NAT database (PlantNATsDB) involving approximately 2 million NAT pairs, covering 69 plant species, including crops and model species with the annotated/curated genome available. PlantNATsDB provides a user-friendly web interface to facilitate the NATs presentation, which, together with a graphical network browser, displays some very complex networks, serving as a reference database to investigate the regulatory function of NATs.

Concerning NAT genesis, researchers have studied it mostly in mammals. Besides the antisense genes that naturally overlap with the sense genes, there are reports of specific situations that can originate such molecules. For example, changes in the regions connecting the UTRs of the locus TP53BP1 NAT-76P were reported by Yelin et al. [31]. The primary transcript of the gene TP53BP1 presents 6.3 kb, having no potential to overlap with *76P *transcripts. The transcript can overlap only when a less abundant 10.5-kb alternatively polyadenylated *TP53BP1 *transcript (or a longer 6.8-kb alternatively polyadenylated *76P *transcript) is generated. Similarly, transcripts with alternative start sites may generate head-to-head overlaps. Additionally, the identification of 48,718 human genes with antisense transcriptional start sites, within transposable elements' sequences [32], revealed that *cis-*NATs may be promoted by these elements.

## 3. NAT Conservation and Distribution

Both NAT loci types (with one or two transcripts with coding capacity) have high positional conservation between different species. This fact was observed by Dahary et al. [33], using an evolutionary approach to analyze the organization of genes with or without NATs. The authors compared human, mouse, and the pufferfish (*Fugu rubripes*) genomes. They found that the NAT loci were twice more likely to preserve their genomic organization throughout vertebrates' evolution than the nonantisense pairs. It implies an overlap existence in the ancestral genome. In reference [34], conducting a study with pseudogenes raised evidence in favor of selective pressure acting on duplicated genes and their *cis-*NATs/*trans-*NATs in specific regions of the Hominoidea genealogy chromosomes. Thus, across species, hundreds of transcribed sense/antisense sequences were probably preserved. It means that they kept the same pattern of overlap and indicates that these transcripts have conserved *in vivo* functions [35]. Likewise, further studies on the origins and mechanisms of regulation, in plants, can use preserved sense/antisense pairs [13]. However, to date, only the report of Wang et al. [13] presents data concerning their conservation in plants (Arabidopsis and rice).

## 4. NAT Coding Capacity

Both transcripts (sense and antisense) from a NAT locus may present a coding capacity or not [16]. However, according to Katayama et al. [12] in mammalian genomes the most common form of interaction between these two molecules is the existence of a sense transcript with coding capacity, interacting with an antisense transcript without the same informational ability. There are some evidence that noncoding RNAs have functionality, even though no coding capacity was associated to them [36]. Ponjavic et al. [36] conducted an evolution study on a set of mouse noncoding RNAs (macroRNAs), comparing their sequences and promoters with human and rat orthologous sequences, under three independent signatures of purifying selection, including substitutions, sequence insertions and deletions, and splicing. 

They pointed that the evolution of that set of noncoding RNAs was not consistent with neutralist explanations. Additionally, according to Jouannet and Crespi [16], noncoding NATs have reduced the sequence conservation when compared to its coding counterpart.

In plants, NAT coding capacity seems to be different. In rice, 86% of the NAT pairs have coding sequence regions in both transcripts [24]. In turn, 82% (1,402 of 1,710) of the predicted Arabidopsis NAT pairs encode for two protein-coding transcripts [37].

## 5. NATs Pairs Expression

Some studies have suggested that *cis*-NAT expression is a transcriptional noise while others claim that such expression is functional. Ling et al. [38] analyzed *cis-*NAT expressions in different tissues and species (human colon, mouse, and rat embryos) in order to settle this impasse. Their assumption was that if orthologous *cis-*NATs showed similar expression patterns when compared with permuted *cis-*NAT pairs, it would provide evidence that *cis-*NATs are actively regulated or subjected to selective pressure. Moreover, the absence of such conservation could favor the transcriptional noise theory. The authors found that the expression patterns of many antisense transcripts are conserved across species. This suggests selective pressure and functionality of these transcripts. Nevertheless, when compared to protein-coding genes, antisense transcripts showed a lower degree of expression conservation in those species. Additionally, they observed a positive correlation between the sense and antisense expression throughout the tissues. 

Further, Zhan and Lukens [37] evaluated protein coding *cis-*NATs (PC *cis-*NATs) expression in Arabidopsis, under a number of different stress conditions and their controls. Then, they compared it with non-*cis-*NAT coding genes (*ncis-*NATs). Their objective was to infer the NAT loci participation in stresses responses, as well as their possible functions. The researchers observed that 83% of PC *cis-*NATs were expressed against around 77% *ncis-*NATs (this difference was significant at *P* < 0.001). They reported that the amount of median *cis-*NAT was substantially higher than that of *ncis-*NAT (*P* < 0.05) in all treatments, but not in the control. Furthermore, PC *cis-*NAT genes were more broadly expressed than *ncis-*NAT genes (around 9% higher). They also suggested that the high transcript abundance of *cis-*NAT genes was due to transcriptional control. 

Lu et al. [39] described another important experiment. They applied high-throughput strand-specific complementary DNA sequencing technology (ssRNA-seq) in rice. Their purpose was to sequence mRNA intensely, in order to assess sense and antisense transcripts stimulated under cold, salt, drought, and normal conditions. They identified 3,358 one-to-one type *cis-*NATs expressed in all conditions. Additionally, there were 2,292 *cis-*NAT pairs detected with both sense and antisense transcripts under normal (1,789 pairs), cold (1,668), salt (1,572), or drought (1,668) conditions. Moreover, the authors characterized the *cis-*NATs response to cold in five subgroups, based on the transcripts' expressional ratio trends. Although all five subgroups presented kinases, some domains and configurations were subgroup specific. This indicates that there is some specialization in NATs activity. For example, sense transcripts of the *cis-*NAT pairs were significantly increased under stress conditions in the subgroup 2, basically formed by proteins with leucine rich repeated domains and glycosyltransferases. In turn, expression levels of the antisense transcripts were greatly reduced in this subgroup. On the other hand, subgroup 3, constituted basically of alpha/beta hydrolase family protein, showed the opposite reaction in *cis-*NAT pairs, with a substantial reduction on the expression levels of sense transcripts under stress conditions and a large rise on the antisense transcripts' expression levels.

## 6. NATs Functions

The NATs performances on their sense transcripts generate distinct biological responses due to the presence of different regulatory mechanisms. Some of these mechanisms require coexpression with their respective target. Others need a time delay between the onset of the antisense and the sense transcription. Additionally, there are other mechanisms manifested in anticorrelated expression patterns of the regulators and their targets. These phenomena have been studied mostly in mammals and unicellular eukaryotes and, according to Lavorgna et al. [40], can be summarized in three main mechanisms.

- Transcriptional Interference: in this mechanism, the regulation occurs not due to the existence of complementarities between the sense and antisense transcripts but because of the act of the concomitant transcription of overlapping genes in opposite directions at the same locus. Thus, the RNA polymerase complexes collide, blocking the rest of the transcription [41]. In an example presented by Prescott and Proudfoot [42], transcription of the *GAL10* and *GAL7* genes occurs at full length when they are rearranged in a convergent orientation. Once the two transcripts begin to overlap, elongation is restricted. The result is a severe reduction in mRNA accumulation.
- RNA masking: the formation of the duplex hybrid structures (dsRNA) resulting from pairing between sense and antisense RNAs may impair the access to existing *cis*-regulatory elements in both transcripts. This hampers the accession of *trans-*acting factors and hinders processes that require protein-RNA interactions such as splicing, mRNA transport, polyadenylation, translation, and degradation. According to Lavorgna et al. [40] this form of steric inhibition could affect any step in gene expression involving protein-RNA interactions, as those mentioned before. One of the most cited examples regards the inhibition of alternative splicing induced by the Rev-ErbA*α* transcript (a NAT), which overlaps one (ErbA*α*2 isoform) of two functionally antagonistic splice forms of the thyroid hormone receptor ErbA*α* mRNA [43]. This mechanism, which shifts the balance between two splice variants, leads to an increase in the ratio of *α*1/*α*2-mRNA levels.
- dsRNA-dependent mechanisms and RNA interference: joint expression of both sense and antisense genes in the same cell may allows the partly overlapping transcripts to bind as dsRNA molecules. This probably will interfere with biological activities linked to the RNA molecules, as mentioned before. However, other mechanisms related to the formation of double-stranded RNA operate in different ways from those described above. The processes of RNA editing and RNA interference are the best known:
  - RNA editing: in this mechanism, RNAs that are completely, or largely, double-stranded experience deamination of adenosine to inosine [44]. The editing of long, perfect RNA duplexes can result in their nuclear retention [45] or cytoplasmic degradation [46].
  - RNA interference (RNAi): the dsRNA formation also generates substrates for RNA interference mechanisms involving DICER-mediated cleavage and small RNA production [47, 48]. Several precedents suggest that sense-antisense transcription can induce gene silencing through an RNAi-dependent mechanism. RNA interference-based antisense effects have been described in plants. Borsani et al. [5] found that the *D*1-pyrroline-5-carboxylate dehydrogenase (P5CDH) antisense overlapping gene pair is a stress-related gene. Once downregulated it leads to the accumulation of proline and to an increase of the ROS production. Even though SRO5 is a gene of unknown function, it generates two types of siRNAs participating in the process of salt tolerance in Arabidopsis. After the dsRNA formation, derived from the connection of the transcripts above, the assembly of two types of siRNA (small interfering RNAs) takes place (one with 24 nucleotides (nt) and another with 21 nt). The siRNA guides the initial cleavage of the P5CDH transcript. This process establishes a phase for the subsequent generation of 21-nt siRNAs by DCL1 (Dicer-like protein 1) and further cleavage of P5CDH transcripts. The NAT-siRNAs downregulate the expression of P5CDH by causing mRNA cleavage. Salt promotes the expression of SRO5, and this induction is required to initiate siRNA formation. In turn, Katiyar-Agarwal et al. [49] reported the detection of nat-siRNAATGB2 which is an endogenous siRNA, specifically induced by the bacterial pathogen *Pseudomonas syringae* carrying effector *avrRpt2. *The authors demonstrated that the biogenesis of this siRNA requires DCL1 and other enzymes. The nat-siRNAATGB2 sequence is complementary to the 3′ UTR region of the sense gene *PPRL.* Thus, it could potentially induce silencing of *PPRL. *This siRNA contributes to *RPS2*-mediated race-specific disease resistance by repressing *PPRL*, a putative negative regulator of the *RPS2* resistance pathway. There are also reports concerning the association of the *cis-*natural antisense siRNAs (*cis-*NAT-siRNAs) with processes other than responses to stress. Ron et al. [50] demonstrated that *cis-*NAT-siRNA-based regulation plays a key role in the Arabidopsis reproductive function. It facilitates gametophyte formation and double fertilization, a developmental process of enormous agricultural value.

In addition to the above mechanisms, gene regulation via the action of NATs may cause induction of DNA methylation with forming dsRNA and subsequent gene silencing. This mechanism acts, for example, in the human hemoglobin 2 gene, resulting in a pathological process called thalassemia [51]. There are also reports associating NATs to the phenomenon of parental imprinting. This is an event in which only one allele (maternal or paternal) is actively transcribed. An evaluation carried out by Katayama et al. [12] estimated that about 80% of the imprinted mouse genes are methilated by such sense/antisense mechanism.

The various mechanisms may also be categorized according to the general molecular processes involved. Considering this classification, Faghihi and Wahlestedt [6] divided them into four primary groups as follows.

- Mechanisms related to transcription: the antisense strand transcription action could modulate the transcription of the sense RNA. In one representation, the RNA synthesis from a DNA strand may hit the transcription of the other strand, suppressing the transcription of antisense RNA sense. This process is called transcriptional collision.
- RNA-DNA interactions: the characteristic of this model would be the interaction between a newly formed RNA transcript, directly or indirectly, with a DNA methyltransferase. It would work as a guide to DNA methylation, which would repress the transcription of sense RNA. Another possibility is the take on of histone and chromatin modifying enzymes by antisense RNA transcripts. This would modulate the chromatin architecture and the epigenetic memory. Genomic imprinting presents itself as yet another situation. The silencing of the X chromosome (X chromosome inactivation) could also be linked to sense/antisense transcripts.
- RNA-RNA interactions in the nucleus: dsRNA formation in the nucleus could result in the transcription of alternative mRNAs. They could end up editing enzymes causing changes in sense RNA transcripts features such as, location, transport, and even stability. In another possibility, sense and antisense RNA could attach to each other. It would cover up processing sites, as well as altering the balance between alternative transcripts.
- RNA-RNA interactions in the cytoplasm: here, the RNA hairpins present in the cytoplasm could act on the sense mRNA stability or even in the translation process. Again, the dsRNAs could hide microRNA (miRNA) binding sites and also function as templates for siRNAs production.

## 7. *In Silico* Identification of NATs

To this date, researchers have identified NATs (*cis* and *trans*) only in model species and relevant crops, such as Arabidopsis, *Lotus japonicus*, rice, sugarcane, and soybean. They have taken advantage of their annotated and curated genome sequences availability and high amount of expressed sequences: ESTs (Expressed Sequence Tags), full-length cDNAs (fl-cDNA), SAGE (Serial Analysis of Gene Expression) tags, MPSS (Massively Parallel Signature Sequencing) tags, RNA-seq, besides others. Despite the ESTs abundance for many plant species, the use of such sequences for NAT identification demands caution. ESTs normally constitute DNA fragments with 300 to 500 bp. They derive from the sequencing of one or both extremities of expressed transcripts under different circumstances [52]. The reads generated from the 5′ end generally include a protein coding sequence, more conserved among species. On the other hand, the 3′ UTR tends to be less conserved [53]. Once sequenced, existing databases provide information to annotate the ESTs. Thus, limitations regarding ESTs reflect the unknown quality of the sequences deposited in public databases, the inadequate or wrong annotations, and the lack of information about the origin/orientation of the sequenced clone. Such imperfections culminate in a questionable reliability of the data [40]. Despite these difficulties, researchers have identified NATs from public EST databases. In such cases, there was a need to develop efficient algorithms to filter out “noisy data” [40]. Chen et al. [23] established criteria to determine the orientation of the human transcripts and indicated ESTs. They confirmed it to be a useful resource to analyze cDNAs from normal and pathologic tissues in diverse developmental stages. Their conclusion was that more than 20% of the human transcripts might constitute *sense*/*antisense* associations. Therefore, this takes place more frequently than expected.

Among the steps for the identification of NAT loci, the sequence orientation is fundamental. In this particular, fl-cDNAs, MPSS- and SAGE-related sequences, and those derived for the strand-specific RNA-seq methodology are the most desirable. The use of fl-cDNAs is adequate due to the presence of the complete 5′ and 3′ end. This facilitates their identification in the genome, permitting the recognition of the respective orientation. SAGE-related sequences are also suitable since their technical protocols establish a tag orientation (5′→ 3′) for the sequencing. Finally, sequences derived from the strand-specific RNA-seq methodology, which is a modified RNA-seq method with an incorporation of deoxy-UTP during a second-strand cDNA synthesis. This induces the uridine-containing strand degradation in the sequencing library, enabling the identification of transcript orientation [54]. When these types of sequences are available and anchored in the genome, the process of analysis and the alignments (Blast) can be carried out in a simple and direct procedure. 

Wang et al. [13] carried out a didactic approach to understand and prospect *cis-*NATs in plants. They developed a methodology for their search in Arabidopsis, using annotated and anchored fl-cDNAs. The researchers applied the following criteria to designate *cis-*encoded natural sense-antisense transcript pairs (NAT pairs): (1) cDNAs of both transcripts can be uniquely mapped to the Arabidopsis genome with at least 96% sequence identity; (2) the two transcripts are derived from opposite strands of the genome; (3) overlapping genomic loci encode both transcript, and the size of the overlaid fragment is longer than 50 nucleotides; (4) the sense and antisense transcripts have distinct splicing patterns. The search resulted in 332 NAT pairs, named cDNA-NATs. Additionally, the authors compared the genomic loci of all Arabidopsis annotated genes. Their aim was to search for gene pairs that overlap in an antiparallel manner (genomic-NATs). A total of 952 putative genomic NATs were identified. The study also demonstrated that only one annotated and anchored transcript composes some cDNA-NATs, indicating that a greater number of NATs may be observed if under other conditions. For expression analysis of NATs, the authors used the data anchored in sense/antisense pairs from MPSS tags as well. Other researchers implemented similar approaches for rice [24, 55] and Arabidopsis [14].

Concerning *trans-*NATs identification, Chen et al. [8] noted that *trans-*NATs may have multiple partners or are imperfect in the RNA bases complementarities. They also relied on the fact that complementary transcripts do not share a common genomic position to adopt the following criteria: (1) the paired region indentified by specific software (DINAMelt) should be coincident with the BLAST-based search and (2) any bubble in such region predicted by the software DINAMelt should be no longer than 10% of it.

### 7.1. NAT Based on Tag Data

SAGE emerged as a potential method to analyze quantitatively, at the same time, numerous transcripts under comparable conditions [56]. Since then, the study of genomes and their transcripts, based on cDNA tags, with lengths of 13–15 nts [57] have used this technique efficiently [56]. It allows the establishment of transcript frequencies in a set of cells [56]. In less complex organisms such as yeast, tags of this size are acceptable. On the other hand, when performing alignments (Blast) in more complex organisms a single tag can align perfectly with more than one transcript, hampering a specific tag-gene association [58]. With the improvement of the method, there was an increase in the tag length in order to make it more reliable. The LongSAGE technique [59] generates 21 bp tags, while the SuperSAGE approach increased this size to 26 bp [9]. 

The SAGE-based techniques allow the revelation and direction of all transcripts, regardless of prior knowledge of them. These methods can detect new RNA transcripts, even the unusual ones or those from alternative splicing [57, 60, 61]. Thus, they can differentiate them as being from the positive (sense) or negative (antisense) strands in the same genome [24].  Table 1 displays a variable number of potential NATs, detected from prospective studies, using SAGE based techniques for model and nonmodel plants species. Usually in a tag annotation, NAT detection is not the primary goal. Occasionally, they are detected accidentally in the course of experiments to approach the sense transcripts using ESTs as mentioned by Werner and Sayer [62]. 

As an example of the data mining steps for identifying NATs, the following *in silico *protocol based on SuperSAGE data from soybean leaves infected with *Phakopsora pachyrhizi* is presented next. This fungus is the etiological agent of ASR, a worldwide disease that causes economic damage in soybean.

## 8. NATs Pairs Expressed in DeepSuperSAGE Libraries of Soybean Infected with *P. pachyrhizi*

### 8.1. SuperSAGE Libraries and Soybean NAT Database

ASR resistant plants (PI561356) in the developmental stage V2 were grown under greenhouse conditions at Embrapa Soybean (Londrina, Brazil). Prior to RNA extraction, the leaves were inoculated with a fungus *P. pachyrhizi* suspension (6 × 10^5^ uredospores·mL^−1^), and collected at 12, 24, and 48 hours after inoculation. The obtained RNAs were subsequently mixed in equimolar amounts to form bulks. This routine aimed to generate the inoculated (PI3T) and the noninoculated mock (PM3T) libraries, as described in Matsumura et al. [63], with samples sequenced (Illumina) with the collaboration of GenXPro GmbH company (Frankfurt, Germany). After *singlets* exclusion (those sequenced just once in either library), the unitags (unique tags) were computed, and those differentially expressed, at the *P* < 0.05 level (Audic-Claverie test performed by DiscoverySpace 4.01 software [64]), were classified as upregulated (UR) or downregulated (DR). Also the unitags were aligned (BlastN; [65]) against the soybean Glyma1 database (*Phytozome* v.9.1; http://www.phytozome.net/soybean; [66]). Only unitag-cDNA alignments with *e-value* ≤ 0.001, score 52 (100% identity covering the 26 bp tag) and the first four bases (CATG) preserved were accepted. The *in silico* mapping of unitags over the NAT pair sequences (*cis* and *trans*) predicted by PlantsNATsDB [8] were considered here.

The PlantsNATsDB v.1.3 database (*Plant Natural Antisense Transcripts Database*; http://bis.zju.edu.cn/pnatdb/; [8]) presents information covering five *cis-*NAT classes, based on their relative orientation and overlap degree [*Divergent* (head to head or 5′ to 5′ overlap), *Convergent* (tail to tail or 3′ to 3′ overlap), *Containing* (full overlap), *Nearby head-to-head* (5′ close to 5′), and *Nearby tail-to-tail* (3′ close to 3′)] and two *trans-*NAT classes (*HC* and *100 nt*). Based on the website information, *cis-*NAT was defined if a pair of transcripts located on opposite strands at adjacent genomic loci showed at least 1 nt overlapping, or their distance on the chromosome was no longer than 100 nts. For *trans-*NAT, the pairs with high sequences complementarities by BlastN analyses followed the criteria: (i) complementary region covered more than one half of the length of either transcript represented a “high-coverage” (HC) *trans-*NAT pair; (ii) transcripts showing a continuous complementary region longer than 100 nts meant a “100 nt” pair. Besides, functional *trans-*NATs should form RNA-RNA duplexes *in vivo*; the PlantsNATsDB authors used DINAMelt software to verify whether the transcript pairs could melt into RNA-RNA duplexes in the complementary regions [8]. 

### 8.2. Unitags Related to Soybean NATs

The unitags BlastN alignments with the soybean transcripts from Phytozome database allowed the identification of the loci/genes involved. Some of them formed the NATs pairs presented in the PlantsNATsDB database. SuperSAGE unitags detected 26,793 of the 77,903 predicted *trans*-NAT pairs (around 1/3) in both sequences of each pair. Concerning *cis*-NAT, 126 pairs of the 436 predicted (also around 1/3 of the total) showed unitags anchored in both sequences of each pair.  Table 2 shows the numbers of *cis*-NAT pairs observed for each class in each chromosome. Comparing these amounts with those from the PlantsNATsDB database, the same one third was observed. Granted that there were exceptions to the *cis*-NAT classes *Divergent* (5/51) and *Containing* (14/119), in which were observed only about 1/10 (observed/predicted NAT pairs). The regulation of unitags mapped in the NAT-pairs, assigned as upregulated, downregulated, or constitutive (nonsignificant at *P* < 0.05), together with the origin of the unitag (if exclusively found in infected or mock library), and the annotation showed how complex the stress response is.

#### 8.2.1. *Cis-*NAT Classes

**Figure 1:**
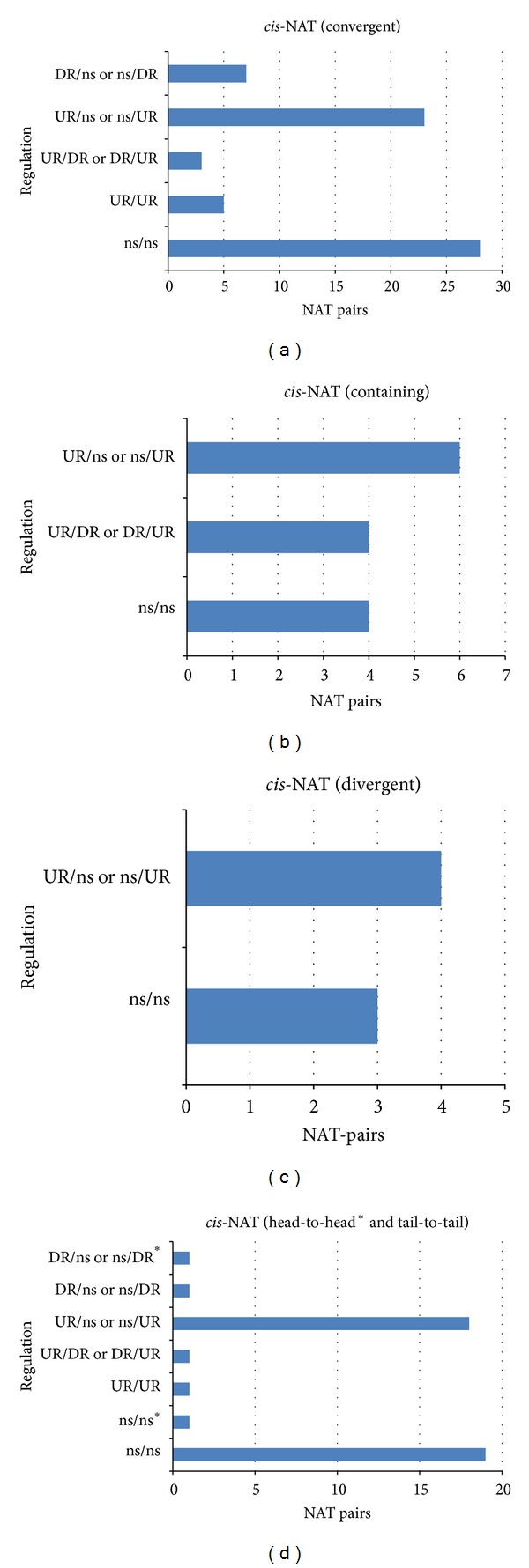
Numbers of *cis*-NAT pairs observed in five classes (PlantsNATsDB database) showing pair regulation based on soybean SuperSAGE unitags mapped in both sequences of each pair. The unitags were originated from library infected with *Phakopsora pachyrhizi* or not and expressed as UR (upregulated), DR (downregulated), or ns (nonsignificant at *P* < 0.05).

*(a) Convergent Class*. This class was the most abundant with 66 *cis-*NAT pairs distributed over the 20 soybean chromosomes. Unitags constitutively expressed (ns) the formed 27 pairs (Figure 1(a)), while the remaining 39 pairs had at least one of the pair differentially expressed. 

Five *cis-*NAT-pairs presented induced unitags (UR, Figure 1(a)) in both sequences of the pair. Examples that can be cited were: thioredoxin superfamily protein (Glyma06g12710; UR)/SEUSS transcriptional coregulator (Glyma06g12720; UR); histidine-containing phosphotransmitter 1 (Glyma13g28390; UR)/inosine-uridine preferring nucleoside hydrolase family protein (Glyma13g28400; UR); erythronate-4-phosphate dehydrogenase family protein (Glyma14g08170; UR)/Sec14p-like phosphatidylinositol transfer family protein (Glyma14g08180; UR). 

For 22 *cis-*NAT pairs, one sequence of the pair presented induced unitags (UR/ns or ns/UR, Figure 1(a)). For example, haloacid dehalogenase-like hydrolase (HAD) superfamily protein (Glyma10g27980; UR)/rotamase CYP 4 (Glyma10g27990; ns); alphamannosidase 3 (Glyma15g10130; UR)/potassium channel in Arabidopsis thaliana 1 (Glyma15g10140; ns); alpha/betaHydrolases superfamily protein (Glyma20g02570; UR)/phloem protein 2-A1 (Glyma20g02580; ns); chaperonin-like RbcX protein (Glyma15g11800; UR)/malonyl-CoA decarboxylase family protein (Glyma15g11810; ns).

Seven *cis-*NAT pairs involved the DR unitag mapped in both sequences of the pairs (DR/ns or ns/DR, Figure 1(a)). It happened to protein kinase superfamily protein (Glyma11g05830; DR)/PLC-like phosphodiesterases superfamily protein (Glyma11g05840); betagalactosidase 5 (Glyma17g37270; DR)/thioredoxin H-type 1 (Glyma17g37280). This last pair was formed by unitags found exclusively in PI3T infected library. Other two NAT pairs also involved unitags exclusively found in the infected library but involved UR unitags [cyclophilin-like peptidyl-prolyl *cis-*trans isomerase family protein (Glyma17g13180)/C3HC zinc finger-like (Glyma17g13190; UR) and phox-associated domain; phox-like; sorting nexin, C-terminal (Glyma07g00690)/Zinc-binding ribosomal protein family protein (Glyma07g00700; UR)]. 

*(b) Containing Class*. A total of 14 *cis-*NAT pairs were identified: 10 of them showing differentially expressed unitags forming the pairs, six of them with induced unitag(s) in one of the pairs (Figure 1(b)). Examples were SAUR-like auxin-responsive protein family (Glyma10g06440; UR)/thylakoid lumen 15.0 kDa protein (Glyma10g06430); plant basic secretory protein (BSP) family protein (Glyma10g40720)/S-methyl-5-thioribose kinase (Glyma10g40730; UR); and sterile alpha motif (SAM) domain-containing protein (Glyma13g44460)/nonannotated Glyma13g44470 (with UR unitag). The last two pairs presented unitags found exclusively in the PI3T infected library. Four other pairs showed divergent regulation (UR and DR forming the pair). For this group, there was no gene/function information available.

*(c) Divergent Class*. Seven *cis*-NAT pairs were identified (Figure 1(c)), and five of them were mapped in soybean chromosomes (Table 2). Four of the seven pairs presented induced unitag(s) in one of the sequences in which two of the pairs were formed basically with unitags only found in the PI3T infected library [haloacid dehalogenase-like hydrolase (HAD) superfamily protein (Glyma07g30070)/protein of unknown function (DUF565) (Glyma07g30060; UR); nonannotated Glyma13g11820/non-annotated Glyma13g11830 (UR)]. In addition, two other pairs presented no differentially expressed unitags found exclusively in the PI3T infected library [pentatricopeptide repeat (PPR) superfamily protein (Glyma09g37140)/biotin/lipoate A/B protein ligase family (Glyma09g37130); ribosomal protein L31 (Glyma13g20190)/ataurora3 (Glyma13g20180)]. 

*(d) Nearby Head-to-Head Class*. Only two *cis-*NAT pairs were identified (Figure 1(d)); just one involved differentially expressed unitags [(nonannotated Glyma11g31890/nonannotated Glyma11g31880 (DR)]. The annotated NAT pair [paralog of ARC6 (Glyma16g33860)/root hair initiation protein root hairless 1 (RHL1)(Glyma16g33850)] showed no unitags with differences in the expression.

*(e) Nearby Tail-to-Tail Class*. A total of 39 *cis-*NAT pairs were identified (Figure 1(d)); 20 of them showed differentially expressed unitags forming the pair, but only two presented repressed unitags. They were FAD/NAD(P)-binding oxidoreductase family protein (Glyma06g05200; DR)/Chaperone DnaJ-domain superfamily protein (Glyma06g05210); cytochrome P450, family 82, subfamily C, polypeptide 4 (Glyma01g38880; UR)/extralarge G-protein 1 (Glyma01g38890; DR, also UR unitag). 

On the contrary, most of the 20 pairs (18, Figure 1(d)) presented at least one induced unitag. Examples included phenazine biosynthesis PhzC/PhzF protein (Glyma13g39580)/histone superfamily protein (Glyma13g39590; UR); arogenate dehydrogenase (Glyma18g02650)/protein of unknown function (DUF399 and DUF3411) (Glyma18g02660; UR); homology to ABI1 (Glyma13g16640)/MAP kinase kinase 2 (Glyma13g16650; both unitags of this pair were found exclusively in (PM3T mock library) chaperonin-like RbcX protein (Glyma09g00950; UR)/malonyl-CoA decarboxylase family protein (Glyma09g00960); replication protein A 1A (Glyma15g19090; UR)/photosystem II subunit X (Glyma15g19100). 

#### 8.2.2. *Trans-*NAT Classes

**Figure 2:**
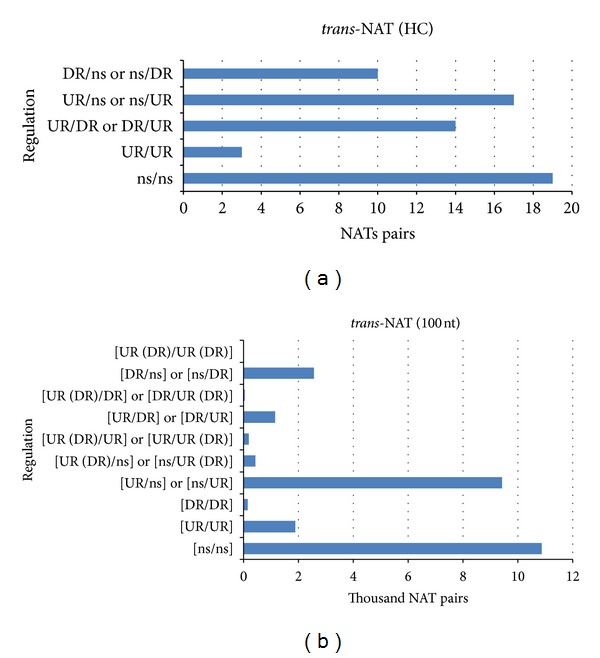
Numbers of *trans*-NAT pairs observed in two classes (HC: “high-coverage”, complementary region covered more than half the length of either transcript; “100 nt”: pair of transcripts showing a continuous complementary region longer than 100 nucleotides; PlantsNATsDB database) showing pair regulation based on soybean unitags mapped in both sequences of each pair. The unitags were from library infected with *Phakopsora pachyrhizi* or not and expressed as UR (upregulated), DR (downregulated), or ns (no-significant at *P* < 0.05).

*(a) HC Class*. In this class, 63 *trans-*NAT pairs were identified; 19 of them presented constitutive unitags (Figure 2(a)). From the 44 pairs with differentially expressed unitags, 14 pairs involved unitags showing opposite regulation (Figure 2(a)), but only two annotated. They were thioredoxin superfamily protein (Glyma04g42080; DR)/SEUSS transcriptional coregulator (Glyma06g12720; UR), and regulator of chromosome condensation (RCC1) family protein (Glyma04g08940; UR)/protein of unknown function (DUF810) (Glyma06g09020; DR). 

Only three *trans-*NAT pairs presented induced unitags in both sequences of the pair (Figure 2(a)), including cytochrome P450, family 82, subfamily C, polypeptide 4 (Glyma01g38880; UR)/ATP binding (Glyma07g09820; UR); SEUSS transcriptional coregulator (Glyma04g42070; UR)/thioredoxin superfamily protein (Glyma06g12710; UR); chalcone and stilbene synthase family protein (Glyma08g11520; UR)/same description (Glyma08g11630; UR). 

Additionally, 17 *trans-*NAT pairs presented induced unitag in one sequence of the pair (Figure 2(a)); for 10 of them both sequences of the pair were nonannotated, but five pairs presented acceptable annotations. They included membrane-associated progesterone binding protein 2 (Glyma04g06340; UR)/DnaJ/Hsp40 cysteine-rich domain superfamily protein (Glyma06g06380; both of the pairs showing unitags exclusively found in PI3T infected library); S-methyl-5-thioribose kinase (Glyma10g40730; UR)/plant basic secretory protein (BSP) family protein (Glyma20g26600; also both of the pairs also showing unitags exclusively found in PI3T infected library); aminoalcoholphosphotransferase 1 (Glyma12g08720; UR)/translocase of the outer mitochondrial membrane 6 (Glyma16g06370).

On the other hand, 10 nonannotated pairs presented repressed unitag mapped in one sequence of each pair (Figure 2(a)); all of these pairs involved chromosome 13. 

*(b) 100 nt Class*. A total of 26,730 *trans*-NAT pairs were identified. From them, 10,867 pairs presented nodifferentially expressed unitags (Figure 2(b)), while 15,863 pairs showed at least one induced or repressed unitag. In this last group, 2,313 pairs were formed by unitags exclusive from the PI3T infected library. On the other hand, 1,326 pairs showed unitags only found in the PM3T mock library. In addition, 117 pairs could be formed by unitags, specifically from one library or the other. Despite these common pairs, the inoculated treatment seemed to promote the expression of more genes, as expected, in response to the stress, when compared with the mock control. To comprehend the regulation of this *trans-*NAT pairs group could be a key aspect in the understanding of ASR plant response.

There were 7,148 *trans*-NAT pairs with at least one sequence of the pair induced and 4,556 with one sequence leastwise repressed. Again, the ASR infection seemed to promote more *trans*-NAT pair formation, and their expressions were more induced than repressed. 

In 2,079, the *trans*-NAT pairs presented induction in both sequences. In addition, 536 of these pairs were formed exclusively by unitags from the PI3T infected library. In turn, there were 74 pairs formed uniquely from the mock library. Finally, 16 pairs could be exclusive formed but with unitags observed in one or the other library. For 192 of the 2,079 pairs, besides the induced unitags observed (in both NAT sequences of the pair), repressed unitags were also observed. This fact increases the combination and complexity of the stress response. 

Conversely, 201 *trans*-NAT pairs showed repressed unitag(s) in both sequences of the pair. Two of them presented alternative induced unitag(s) for both sequences as well [deletion of SUV3 suppressor 1(I) (Glyma06g19530)/mitogen-activated protein kinase 16 (Glyma15g10940) and homeodomain-like superfamily protein (Glyma13g19910)/mitogen-activated protein kinase 16 (Glyma15g10940)]. 

Ultimately, 49 of them also presented alternative induced unitags for just one sequence of the pair. Only two of those 49 pairs were formed with unitags exclusive from the PI3T infected library. However, 23 were formed by unitags uniquely found in the PM3T mock library. A total of 150 *trans*-NAT pairs showed purely repressed unitag(s) in both sequences of the pair. From these, only seven were formed by unitags exclusively from the PI3T infected library against 35 pairs formed with unitags uniquely from the PM3T mock library. Again, the ASR applied stress modified the soybean transcriptome, providing an opportunity to identify potential NAT pairs differentially expressed.

#### 8.2.3. Combining Information from PlantNATsDB, Phytozome, and SuperSAGE Data

As seen in the previous sections, soybean subjected to infection by *P. pachyrhizi* produces thousands of NAT pairs. They all differ from each other in structural configurations (*cis* or *trans*) and possible regulations (e.g., transcribed sense and antisense, induced, repressed, etc.). Since biological information needs to be added to the data, one alternative is combining these expression data with those online available from public databases, such as the PlantNATsDB and Phytozome. From this, a deeper insight into the NAT pairs orchestration can be observed with the Glymas (designation of loci/transcripts predicted for soybean, [66]). The expression analysis of the Glyma10g40730 locus (for s-methyl-5-thioribose kinase) represents an example of this. It generates transcripts that form a *cis*-NAT with the expression product of Glyma10g40720 locus, which is a plant basic secretory protein (BSP). Based on unitags, the Glyma10g40730 was induced (alternative transcript Glyma10g40730.2), and the Glyma10g40720 was not differentially expressed (*P* < 0.05) after stress submission. Additionally, this NAT pair was only expressed in the infected library (Section 8.2.1(b)). This suggests a functioning role in the stress response studied here, where the NAT pair action mechanism (see item 6) needs to be determined.

**Figure 3:**
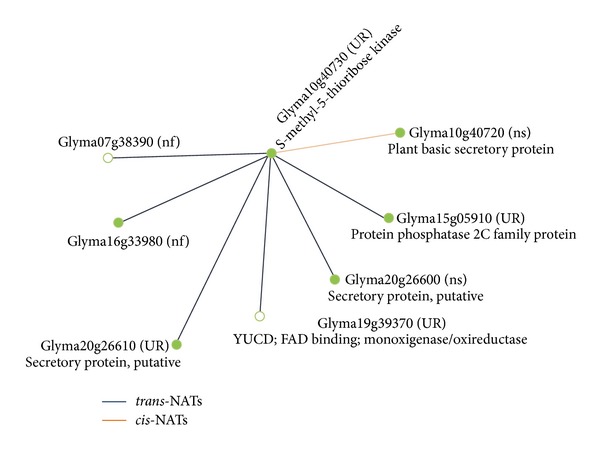
NAT pairs (*cis* and *trans*) involving soybean Glyma10g40730 and others transcripts, in association with the regulation observed with SuperSAGE unitags mapped in the respective transcripts. Adapted from the Network Viewer/PlantNATsDB database. UR: upregulated (in soybean leaves infected with *Phakopsora pachyrhizi versus* mock control); ns: no differentially expressed (*P* < 0.05); nf: not found. Green circles: NATs that do not have another antisense relationship with any other locus. White circle: NATs that have other antisense relationships.

Detailed analysis of the Glyma10g40730 locus complementary relationship in the soybean genome showed that it can form both NATs types (Figure 3 and Table 3). Besides forming a *cis*-NAT pair with the Glyma10g40720 locus, as already mentioned, the Glyma10g40730 forms *trans*-NAT pairs with other six loci located on five different chromosomes (Figure 3 and Table 3), all of them having coding properties (data not shown). In Table 3, it is observed that the expression product of a sense acting Glyma in a situation can become antisense, depending on its NAT partner. Such data may be seen in “Gene A”, interpreted as sense and “Gene B”, as antisense.

**Figure 4:**
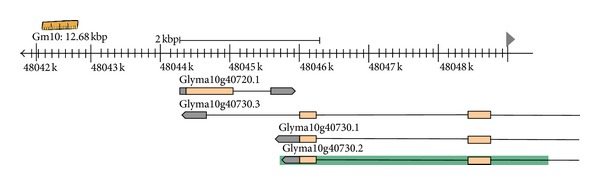
*cis*-NAT pair involving Glyma10g40730 and Glyma10g40720 transcripts. Adapted from Phytozome website (http:// www.phytozome.net/).

It is worth noting that the Glyma10g40730 locus supposedly encodes three alternative isoforms codifying transcripts of the s-methyl-5-thioribose kinase enzyme. They all have the potential to form NAT pairs with the single transcript produced by the Glyma10g40720 locus (Figure 4). This suggests that the expression of all the isoforms may be regulated by NAT pairing. Further, the considered sense transcript expression (12.5 tags per million) was higher than that of its respective antisense (2.5 tags per million). Such expression configuration has been seen in other situations at different rates. Ling et al. [38], analyzing NATs in different human, mouse, and rat tissues, observed that the average expression of antisense transcripts is lower than that of sense transcripts at both the intron and exon level. Lembke et al. [75] also reported this situation in sugarcane subjected to drought.

The data set reported here shows precise relationships between the transcripts acting in pairs. This represents a better understanding of the soybean transcriptome infected by a fungus (*P. pachyrhizi*) since the genes are not considered independently. Thus, specific pathways can be studied so that a more comprehensive picture of the gene orchestration can be obtained.

## 9. Concluding Remarks

This review presented some important features relative to NATs. It covered genesis, functions, and capacity of regulating the expression of target genes at different levels. It also showed that the understanding of NAT pair expressions and their participation in the complex network involving plant response to abiotic or biotic stresses are relevant for the development of future interventions in organisms' metabolism by plant breeders. In this way, the *in silico* NAT pair identification represents the first step for this accomplishment. 

Here, the deepSuperSAGE technique was combined with bioinformatics tools, allowing identifying soybean transcripts involved in NAT pairs, predicted before in the PlantsNATsDB database. Based on expression of unitags from leaves infected with *P. pachyrhizi*, the analysis showed that around 1/3 of the predicted *cis*- and *trans*-NAT were detected with unitags expressed in both sequences of each pair. For the unitags mapped in NAT-pairs, respective regulation (UR, DR, or ns, at *P* < 0.05) were assigned, as well as the origin of the unitag (if they were exclusively found in the infected or in the mock library) and the unitag annotation. This approach considered the transcriptome in a more comprehensive way. The understanding of NATs regulation and relationships could be a key aspect in the plant response to stresses, besides being a source of study for future works.

## Figures and Tables

**Table 1:** NAT prospection in plant studies using SAGE or derivatives techniques.

Specie	Unitags	NATs	Library	Techique	Reference
Rice	15131	3*	Mature leaf; immature seed tissue	SG	[67]
*Lotus japonicus *	8532	25**	Nodulating roots	SG	[68]
Sugarcane	5227	894	Mature leaves	SG	[69]
Wheat	29261	5*	Seed (developing wheat caryopsis)	SG	[70]
Barley	41909	6*	Seed (during the malting process)	SG	[71]
*Arabidopsis *	26456	5555	Seedlings/low temperature	SG	[72]
Rice	83382	6050	*Magnaporthe grisea* infection	LS	[73]
Potato	22233	6*	Tubers at the end of flowering	LS	[74]
Wheat	37615	845	Grains (hot/dry conditions)	LS	[60]
*Brassica napus *	32395	309	Seed (23 DAP/35 DAP)	LS	[67]
Chickpea	17493	170	Root/drought	SS	[68]
*Solanum torvum *	34269	891	Cadmium-stressed roots	SS	[69]

*Considering the 100 most abundant tags; **considering the differentially expressed tags; SG: SAGE; LS: LongSAGE; SS: SuperSAGE.

**Table 2:** Number of soybean genes (loci by chromosome) with unitags SuperSAGE mapped in both sequences of the *cis-*NAT pairs of five classes.

Chrom.	Convergent	Divergent	Containing	Nearby tail-to-tail	Nearby head-to-head
1	1	0	0	2	0
2	8	0	0	4	0
3	3	1	0	0	0
4	5	0	0	2	0
5	4	0	0	0	0
6	2	1	1	5	0
7	4	1	0	1	0
8	3	0	0	3	0
9	2	0	0	2	0
10	2	0	2	1	0
11	4	0	1	7	1
12	3	0	0	3	0
13	8	2	9	2	0
14	2	0	0	1	0
15	4	0	0	1	0
16	1	0	0	0	1
17	5	0	0	2	0
18	1	0	0	1	0
19	2	0	0	1	0
20	2	0	1	1	0

Total	66	5	14	39	2

**Table 3:** NAT pairs (*cis* and *trans*) involving Glyma10g40730 (as gene A or B), with the overlapping (start-end) covering both sequences, according to PlantNATsDB.

Gene A	Start	End	Gene B	Start	End	NAT type	Overlap
Glyma10g40720	1	1410	Glyma10g40730	5411	6820	*cis *	1410
Glyma10g40730	6071	6428	Glyma16g33980	8780	9137	*trans *	358
Glyma07g38390	1606	1737	Glyma10g40730	876	1007	*trans *	132
Glyma10g40730	6069	6424	Glyma20g26610	381	736	*trans *	356
Glyma10g40730	964	1069	Glyma19g39370	1716	1821	*trans *	106
Glyma10g40730	6073	6740	Glyma20g26600	180	835	*trans *	668
Glyma10g40730	959	1070	Glyma15g05910	2440	2551	*trans *	112
